# Effect of Health Insurance on Hepatitis C Sustained Virologic Response Rates to Sofosbuvir-Based Treatment Regimens in a South Florida Community Hospital

**DOI:** 10.1177/2325958219835590

**Published:** 2019-03-29

**Authors:** Paula Eckardt, Jianli Niu, Angela Savage, Tara Griffin, Elizabeth Sherman

**Affiliations:** 1 Division of Infectious Diseases, Memorial Physician Group, Hollywood, FL, USA; 2 Office of Human Research, Memorial Healthcare System, Hollywood, FL, USA; 3 Nova Southeastern University College of Pharmacy, Fort Lauderdale, FL, USA

**Keywords:** chronic hepatitis C, HIV, SVR, sofosbuvir-based regimens, insurance status

## Abstract

The high cost of direct-acting antiviral–based regimens raises concerns about the outcome of treatment in uninsured patients with chronic hepatitis C virus (HCV) infection. This study assessed the relationship between health insurance status and sustained virologic response (SVR) rates in a community hospital in South Florida. Sofosbuvir-based therapy was initiated in 82 patients, of which 73% were uninsured and 28 (34%) were HIV coinfection. The overall SVR rate for those tested was 98%. The SVR rates were similar between HCV mono- and HCV/HIV coinfected patients (96% versus 100%, *P* = .204). Uninsured patients, with access to patient assistance programs, had comparable SVR rates to insured patients (100% versus 95%, *P* = .131). However, there was a trend toward a higher rate of loss to follow-up in uninsured compared to insured patients (25% versus 9%, *P* = .116). Strategies specific to adherence to treatment for uninsured patients are needed to reduce rates of loss to follow-up.

**What Do We Already Know About This Topic?**

The reported higher sustained virologic response (SVR) rates with direct-acting antiviral agent (DAA)-based regimens were mainly derived from clinical trials treating well-insured populations. Their high cost raises concerns about the outcome of treatment in patients who are socioeconomically disadvantaged and underinsured.

**How Does Your Research Contribute to the Field?**

This study demonstrates that uninsured patients with chronic hepatitis C virus (HCV) infection, with access to patient assistance programs, can achieve comparable SVR rates seen from clinical trials. Uninsured patients showed a trend to a higher rate of loss to follow-up for documentation of SVR.

**What Are Your Research’s Implications toward Theory, Practice, or Policy?**

Effective outcomes with DAA-based therapy can be achieved in underinsured populations with access to patient assistance programs. Strategies specific to adherence to treatment and retention in care for uninsured patients are needed to reduce rates of loss to follow-up.

## Introduction

Hepatitis C virus (HCV) remains a leading cause of chronic liver disease worldwide, and genotype 1 HCV is the most common infection, accounting for approximately 68% of all HCV infections in the United States.^1^ The treatment of choice for patients with genotype 1 HCV infection has rapidly evolved in recent years, with improved sustained virologic response (SVR) rates and reduced HCV-related morbidity and mortality.^2,3^ A combination of direct-acting antiviral agents (DAAs) are increasingly used for the treatment of HCV genotype 1, as they are highly efficacious, have good tolerance, and provide a shortened treatment duration compared to conventional therapy with peginterferon (PegIFN) plus ribavirin.^4-6^

Sofosbuvir (SOF) is a pyrimidine nucleotide analogue that inhibits HCV NS5B polymerase required for viral replication.^7^ SOF has shown high efficacy in combination with several other drugs, with or without PegIFN, against HCV, with SVR rates of 95% to 99% among treatment-naive patients and 94% in previously treated patients.^4-6,8^ SOF-based regimens have also been reported to markedly improve SVR rates in HIV/HCV coinfected patients.^9,10^ However, the reported higher SVR rates with DAA-based regimens were mainly derived from clinical trials conducted in academic centers treating well-insured populations.^4-6,9,10^ These trials did not include underserved minority populations, such as homeless persons, substance users, and persons with mental health disorders. Patients without health insurance may have to pay all or most prescription drug costs, likely leading to skipped doses or unfilled prescriptions. Thus, the overall efficacy of DAA-based regimens on treatment of HCV in resource-limited settings remains uncertain.

In 2015, Memorial Healthcare System in Hollywood, Florida, facilitated patient assistance program (PAP) enrollment through pharmaceutical company sponsorship to allow lower income people who are uninsured or underinsured and who do not qualify for insurance programs such as Medicaid or Medicare to access free medication. The PAPs have the potential to increase patients’ access to needed medications. We therefore sought to evaluate SVR rates of patients with HCV infection treated with SOF-based regimens in a community hospital in South Florida and to determine whether there is an association between health insurance status and SVR. In addition, baseline clinical characteristics and their association with SVR rates were assessed.

## Methods

### Study Population and Setting

This is an observational retrospective study from Memorial Regional Hospital of the Memorial Healthcare System, Hollywood, Florida. All patients with HCV infection who had been treated with SOF-based regimens in the outpatient practice between January 2005 and April 2017 were included. Treatment regimens and dosages were selected by the physician following the evidence-based guidelines (AASLD-IDSA. Recommendations for testing, managing, and treating hepatitis C. https://www.hcvguidelins.org). Treatment regimens during the study period included (1) SOF + ledipasvir, (2) SOF + daclatasvir, (3) SOF + simeprevir, (4) SOF + ribavirin, (5) SOF + ribavirin + PegIFN, or (6) SOF + ledipasvir + ribavirin. Pharmacists completed prior authorization paperwork for insured patients and applications for PAP through pharmaceutical companies for uninsured patients. Complications, adverse effects, medication adherence were monitored, and nurse practitioners scheduled follow-up appointments and laboratory testing for complete blood counts, CD4 levels (for HIV coinfected patients), renal and hepatic function panels, and HCV RNA, every 4 weeks during treatment, at the end of treatment, and 12 weeks after the end of treatment to assess for SVR.

### Data Collection

The research nurses extracted data from electronic medical records into an Excel (Microsoft) spreadsheet. Participants were categorized as insured or uninsured patients according to information collected at treatment initiation. SVR was defined as the absence of quantifiable HCV RNA in serum (<15 IU/mL) at 12 weeks after the end of treatment. Patients were defined as loss to follow-up if they did not visit the clinic for SVR assessment after the end of treatment. Viral relapse was defined as undetectable HCV RNA at the end of treatment but subsequent detectable HCV RNA at 12 weeks after the end of treatment. Age, gender, associated diabetes, alcohol abuse, HCV genotype, HIV-coinfection, hepatitis B virus coinfection, baseline HCV RNA, liver fibrosis stages (as classified by METAVIR fibrosis scoring system^11^), prior treatment failure, serum alanine transaminase (ALT) and aspartate aminotransferase (AST), CD4 levels (for HIV coinfected patients), and insurance status were analyzed for predictors of SVR.

This study was approved by the institutional review board at the Memorial Healthcare System (approval no. MH2017.028). This committee waived the need to obtain consent for the collection, analysis, and publication of the retrospectively obtained and anonymized data for this study.

### Statistical Analysis

Categorical variables were summarized by the number of patients and percentages of their group and continuous variables by medians and interquartile ranges (IQR). Proportions were compared using the χ^2^ or Fisher exact test. Odds ratios (OR) and 95% confidence intervals (CIs) of the proportion of patients who achieved SVR were calculated. SVR across subgroups based on insurance status was compared using the χ^2^ test or Fisher exact test. All tests were 2 tailed, and a *P* < .05 was considered significant for all tests. Univariate logistic regression was performed independently for each candidate covariate (gender, HCV RNA, HCV/HIV coinfection, fibrosis, ALT, AST, and health insurance status), and the covariates found significantly predicting SVR (*P* < .05) on univariate analysis were entered into multivariate analysis with potential confounders to identify independent factors predicting SVR. All statistical analyses were performed using SPSS version 25 (IBM Corp, Armonk, New York), and graphs were generated using GraphPad Prism 7.0 for Windows (GraphPad Software, San Diego, California).

## Results

SOF-based therapy was initiated in 82 patients. Median age was 56 (IQR 49-59) years, and the majority (57%) were male. The most common HCV genotype was genotype 1 (80%), 11 (14%) had genotype 3, and 5 (6%) had genotype 2. Among the risk factors for HCV acquisition, 7 (9%) patients were infected post-blood transfusion, 26 (32%) were intravenous drug users, and the remaining 49 (59%) patients had no known etiology. Forty-two (51%) patients were classified as having stage F3-4 liver fibrosis, and 40 (49%) were classified as having stage F0-2 liver fibrosis. Most patients were treatment naive with 7 (9%) patients with a history of previous treatment failure. HIV coinfection was observed in 28 (34%) patients. Hepatitis B virus coinfection was found in 4 (5%) patients. Common comorbidities included 22 (27%) patients who had a history of diabetes, 15 (18%) patients who reported a history of alcohol/drug use, and 16 (20%) patients who had a history of mental health disorders. The median of pretreatment viral load was 2.4 × 10^6^ IU/mL, with an IQR of 0.8 to 5.9 × 10^6^ IU/mL. Median ALT and AST levels were 66 (IQR: 44-109) U/L and 57 (IQR: 31-89) U/L, respectively. Among 82 patients, 60 (73%) were uninsured and 22 (27%) were covered by medical insurance. Insured patients were more likely to be male (77% versus 50%, *P* < .05) and coinfected with HIV (73% versus 20%, *P* < .05) compared to the uninsured. No significant differences in age, risk factor for infection, HCV genotype, HCV RNA, stage of liver fibrosis, baseline levels of ALT and AST, or history of diabetes or alcohol/drug use were observed between uninsured and insured patients (Table 1).

**Table 1.** Baseline Characteristics of the Study Population.

Variables	Overall	Uninsured	Insured	*P* Value
	N = 82	n = 60	n = 22	
Gender, male (%)	47 (57%)	30 (50%)	17 (77%)	.042
Age, years, median (IQR)	56 (49-59)	56 (48-60)	56 (50-59)	.504
HCV genotype				
1	66 (80%)	49 (82%)	17 (77%)	.754
2	5 (6%)	4 (7%)	1 (5%)	1.000
3	11 (14%)	7 (11%)	4 (18%)	.475
HCV RNA, ×10^6^ IU/mL				
Median, IQR	2.4 (0.8-5.9)	2.8 (0.9-6.2)	2 (0.4-4.8)	NS
Source of HCV infection, n (%)				
Blood transfusion	7 (9%)	6 (10%)	1 (5%)	.668
Intravenous drug use	26 (32%)	21 (35%)	5 (23%)	.422
Unknown	49 (59%)	33 (55%)	16 (72%)	.204
Fibrosis stage (METAVIR)				
F0-F2	40 (49%)	30 (50%)	10 (46%)	.805
F3-F4	42 (51%)	30 (50%)	12 (54%)	.805
Prior HCV treatment failure	7 (9%)	6 (10%)	1 (5%)	.668
HIV coinfection	28 (34%)	12 (20%)	16 (73%)	.0002
Chronic HBV	4 (5%)	2 (3%)	2 (9%)	.291
Diabetes	22 (27%)	16 (27%)	6 (27%)	1.000
History of alcohol/drug use	15 (18%)	11 (18%)	4 (18%)	1.000
Biochemical analysis, U/L				
ALT, median (IQR)	66 (44-109)	68 (48-109)	60 (37-99)	.375
AST, median (IQR)	57 (31-89)	61 (31-90)	53 (30-88)	.938

Abbreviations: ALT, alanine transaminase; AST, aspartate aminotransferase; IQR, interquartile range; HBV, hepatitis B virus; HCV, hepatitis C virus; NS, not significant.

**Figure 1.**
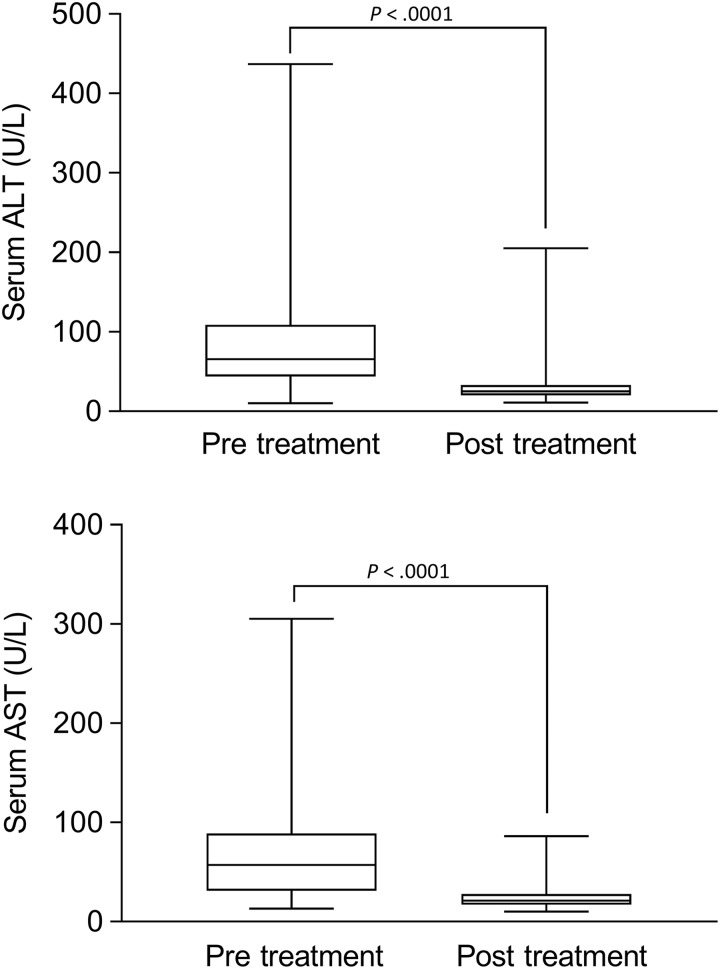
Biochemical response to antiviral treatment. The increased levels of serum alanine transaminase (ALT) and aspartate aminotransferase (AST) pretreatment were significantly reduced and returned to normal levels in patients who received treatment with sofosbuvir (SOF)-based regimens.

As illustrated in Table 2, the most common treatment regimens were SOF + ledipasvir (n = 52, 63%), followed by SOF + ribavirin (n = 11, 21%), SOF + daclatasvir (n = 9, 11%), and SOF + ribavirin + PegIFN (n = 6, 7%; Table 2). Overall, 72 (88%) patients completed a full course of treatment. By 12 weeks after the end of treatment, SVR could be tested in 65 patients, and 64 (98%) achieved SVR (95% CI: 0.92-0.99). One patient with HIV coinfection completed HCV therapy but relapsed at the time of SVR assessment. 17 (21%) of 82 patients were lost to follow-up. Patients who achieved SVR had documented biochemical responses with normalized ALT and AST tests (Figure 1).

**Table 2.** Treatment Outcomes by HCV SOF-Based Regimens.

Treatment Regimens	Patients, n = 82	HCV Genotype	Treatment Outcomes	
			SVR, %	Lost to Follow-up, n
Treatment-naive				
Sofosbuvir + ledipasvir	49	1a, 1b	98	14
Sofosbuvir + daclatasvir	9	3a	100	2
Sofosbuvir + simeprevir	3	1a, 1b	100	0
Sofosbuvir + ribavirin	9	1a, 2, 3a	100	1
Sofosbuvir + ribavirin + PegIFN	5	1a, 1b	100	0
Treatment experienced				
Sofosbuvir + ribavirin	2	2	100	0
Sofosbuvir + ribavirin + PegIFN	1	1a	100	0
Sofosbuvir + ledipasvir	3	1	100	0
Sofosbuvir + ledipasvir + ribavirin	1	1a	100	0

Abbreviations: HCV, hepatitis C virus; PegIFN, peginterferon; SOF, sofosbuvir; SVR, sustained virologic response.

All HIV/HCV coinfected patients were treated simultaneously with antiretroviral therapy. All of the patients had CD4 counts above 200 cells/mm^3^ (median: 461; IQR: 337-589) at treatment. Of 28 HCV/HIV coinfected patients, 15 (54%) patients were treated with SOF + ledipasvir, followed by SOF + ribavirin (n = 5, 21%), SOF + daclatasvir (n = 3, 11%), SOF + ribavirin + PegIFN (n = 3, 11%), and SOF + simeprevir (n = 2, 7%). SVR could be tested in 25 patients, and 3 patients were lost to follow-up. SVR occurred in 24 (96%) of 25 patients (95% CI: 0.80-0.99), and 1 patient mentioned earlier completed HCV therapy with SOF + ledipasvir but relapsed at the time of SVR assessment. The rates of SVR were similar in HIV/HCV coinfected patients compared to HCV mono-infected patients (96% versus 100%, *P* = .204).

As shown in Table 3, of 60 uninsured patients treated with SOF-based regimens, SVR could be tested in 45 patients, and 15 (25%) patients were lost to follow-up. SVR was achieved in 45 (100%) of 45 patients (95% CI: 0.92-1.0). Of the 22 insured patients, SVR could be tested in 20 patients, and 2 (9%) patients were lost to follow-up. SVR was achieved in 19 (95%) of 20 patients (95% CI, 0.76-0.99). There was no difference in SVR rates in insured and uninsured patients (95% versus 100%, *P* = .131). Rate of loss to follow-up was slightly higher in uninsured than in insured, but this difference was not statistically significant (25% versus 9%, *P* = .116).

**Table 3.** Treatment Outcomes by Patient Health Insurance Status.

Health Insurance Status	Patients, n	Treatment Outcomes	
		SVR, n (%)	Loss to Follow-up, n (%)
Uninsured	60	45/45 (100)	15 (25)
Insured	22	19/20 (95)	2 (9)

Abbreviation: SVR, sustained virologic response.

By univariate analyses, compared to insured, uninsured patients who enrolled in PAP had similar SVR rates (odds ratio [OR] 2.96, 95% CI: 0.61-14.29, *P* = .178; Table 4). Other covariates, such as gender, HIV coinfection, liver fibrosis stage, HCV RNA, ALT, and AST, showed no association with SVR rates (Table 4). We then adjusted for potential confounders including these factors using multivariate analyses. After adjustment, a multivariate analysis revealed that SVR rates were similar between uninsured and insured patients (OR: 1.67, 95% CI: 0.24-11.61, *P* = .606; Table 5). These adjusted analyses confirm that insurance status was not associated with SVR rates of SOF-based regimens.

**Table 4.** Univariate Analysis of Association of SVR with Baseline Characteristics.

Variables		OR	95% CI	*P* value
Gender	Male versus Female	1.46	0.48-4.39	.499
Coinfection	HCV versus HCV/HIV	2.6	0.67-10.06	.166
Fibrosis stage	F0-2 versus F3-4	3	0.87-10.29	.081
HCV RNA, × 10^6^ IU/mL	<2.4 versus >2.4	1.46	0.48-4.39	.499
ALT	Normal versus elevated ALT	2.01	0.66-6.07	.218
AST	Normal versus elevated AST	2.63	0.86-8.05	.089
Health insurance status	Uninsured versus Insured	2.96	0.61-14.29	.178

Abbreviations: ALT, alanine transaminase; AST, aspartate aminotransferase; CI, confidence interval; HCV, hepatitis C virus; IQR, interquartile range; OR, odds ratio; SVR, sustained virologic response.

**Table 5.** Multivariate Analysis of Association of SVR with Baseline Characteristics.

Variables		OR	95% CI	*P* value
Gender	Male versus female	0.48	0.11-2.08	.328
Coinfection	HCV versus HCV/HIV	3.53	0.61-20.38	.159
Fibrosis stage	F0-F2 versus F3-F4	2.36	0.51-11.04	.274
HCV RNA, × 10^6^ IU/mL	<2.4 versus >2.4	1.78	0.50-6.34	.37
ALT	Normal versus elevated	2.11	0.41-11.11	.378
AST	Normal versus elevated	1.56	0.34-7.24	.571
Health insurance status	Uninsured versus insured	1.67	0.24-11.61	.606

Abbreviations: ALT, alanine transaminase; AST, aspartate aminotransferase; CI, confidence interval; HCV, hepatitis C virus; IQR, interquartile range; OR, odds ratio; SVR, sustained virologic response.

## Discussion

Although SOF-based regimens have higher rates of SVR in treating well-insured populations,^4-6^ it remains uncertain whether the results will directly translate to patients who are uninsured with limited access to HCV diagnostic and treatment services. In this study, we evaluated HCV treatment outcomes in uninsured and insured patients at a community hospital in Hollywood, Florida, USA. Overall, 88% completed a full course of treatment. SVR could be tested in 65 patients, and the overall SVR rates (98%) were comparable to those reported in clinical trials (95% and higher). Uninsured patients who participated in PAP achieved similar rates of SVR compared to insured patients (100% versus 95%, *P* = .131). There were no differences in SVR rates by insurance status, sex, HIV coinfection, liver fibrosis stage, HCV RNA, history of diabetes, or history of alcohol/drug use. However, there was a trend toward higher rates of loss to follow-up in uninsured patients (*P* = .116).

Limited studies report the efficacy of SOF-based therapy in socioeconomically disadvantaged patients.^12,13^ Beck et al^12^ reported an SVR rate of 93% in 189 patients receiving SOF-based therapy, in which 95% of patients were insured. Yek et al^13^ reported SVR was achieved in 90% of patients, with 56% of patients being uninsured and 13% covered by Medicaid. Our study patients are unique in that it consists of a large group of uninsured patients (73%) when compared to previous studies.^12,13^ SVR rates in those tested patients were comparable to those reported in clinical trials treating well-insured populations.^4-6^ This finding is consistent with earlier research that has demonstrated effective outcomes with SOF-based therapy in socioeconomically disadvantaged populations.^14-16^ The number of Americans who cannot afford to pay for needed prescription medications is on the rise, and these patients also tend to skipped doses or unfilled prescriptions due to cost concerns. Patient assistance programs have become an increasingly important part of quality medical care for patients who lack health insurance or prescription drug coverage. Our findings are consistent with the results of DeBose-Scarlett et al^17^ who investigated the outcome of HCV treatment regimens (DAAs) in uninsured patients showing that insured and uninsured patients with chronic HCV infection, with access to PAPs, can be treated and have comparable clinical outcomes.

Several clinical variables that may explain different response to antiviral treatment among patients with HCV infection have been suggested.^18-21^ By univariate and multivariate analyses, we found no relationship between patients’ baseline characteristics and SVR rates. These data provide further evidence that baseline characteristics (such as HCV genotype, sex, fibrosis status, HIV coinfection, prior treatment failure, and HCV RNA) have less impact on SVR rates with DAA-based antiviral therapy.^15,20,21^ However, rates of loss to follow-up in uninsured patients were slightly higher than in insured patients (25% versus 9%, *P* = .116), which is higher than the 5% to 15% reported in previous studies in indigent populations.^15,21^ Recently, DeBose-Scarlett et al^17^ reported that there were still a significant number of patients who were prescribed and initiated treatment but did not ultimately follow through to the end of the HCV care cascade. When comparing our results to those of recent studies,^14-17^ it must be pointed out that future studies should develop adapted models of care for populations living with HCV to ensure their adherence to antiviral therapy and retention in medical care.

Studies on the efficacy and safety of SOF-based regimens in HIV/HCV-coinfected patients have been reported, with overall SVR rates ranging from 91% to 98%, regardless of liver fibrosis stage and treatment experience.^8,9,22^ In our study, SVR rates in HIV coinfected patients, including patients with prior treatment failure and those with cirrhosis, were almost the same as those observed in HCV mono-infected patients (96% versus 100%, *P* = .204). The observed SVR rate of 96% in these HIV coinfected patients is consistent with those previously reported in patients with HIV coinfection,^8,22^ thus suggesting an equal response to the SOF-based regimens in HCV mono-infected and HCV/HIV coinfected patients. This is contradicted by a recent prospective multicohort study, where Neukam et al^23^ reported that HIV/HCV coinfected patients have a worse response to DAA-based therapy than HCV mono-infected individuals (86.3% versus 94.9%, *P* = .002). The reasons for this discrepancy are uncertain. Factors such as drug interactions, comorbidities, race/ethnicity, and HCV genotype have been shown to affect treatment outcomes. For example, lower SVR rates in patients infected with HCV genotype 3 have been described previously,^20^ and patients of black race had lower SVR rates compared with patients of white race.^16,24^ In our study, the majority of these HIV coinfected patients were infected with HCV genotype 1 (86%). The limited number of patients and the variations of HCV genotype in the present study may account for the above described differences in findings and larger studies are needed to confirm these observations.

There are a few limitations to our study. First, this was a retrospective observational study, with the inherent limitations with a study of this design. Also, we cannot state with certainty that the high SVR rates we had are directly comparable to the general population, as we did not have a sufficient number of patients to fully examine SVR rates of the different SOF-based regimens. In addition, SVR in patients who were lost to follow-up before obtaining their SVR testing is unknown.

In conclusion, this study assessed outcomes with SOF-based regimens in socioeconomically disadvantaged populations and demonstrates uninsured patients with access to PAPs have similar HCV treatment outcomes as insured patients. There was a trend toward higher rates of loss to follow-up in uninsured compared to insured patients. Future development of adapted models of care for HCV populations in our community is needed to ensure their adherence to antiviral treatment and retention in medical care.
